# How learning to abstract shapes neural sound representations

**DOI:** 10.3389/fnins.2014.00132

**Published:** 2014-06-03

**Authors:** Anke Ley, Jean Vroomen, Elia Formisano

**Affiliations:** ^1^Department of Medical Psychology and Neuropsychology, Tilburg School of Social and Behavioral Sciences, Tilburg UniversityTilburg, Netherlands; ^2^Department of Cognitive Neuroscience, Faculty of Psychology and Neuroscience, Maastricht UniversityMaastricht, Netherlands

**Keywords:** auditory perception, perceptual categorization, learning, plasticity, MVPA

## Abstract

The transformation of acoustic signals into abstract perceptual representations is the essence of the efficient and goal-directed neural processing of sounds in complex natural environments. While the human and animal auditory system is perfectly equipped to process the spectrotemporal sound features, adequate sound identification and categorization require neural sound representations that are invariant to irrelevant stimulus parameters. Crucially, what is relevant and irrelevant is not necessarily intrinsic to the physical stimulus structure but needs to be learned over time, often through integration of information from other senses. This review discusses the main principles underlying categorical sound perception with a special focus on the role of learning and neural plasticity. We examine the role of different neural structures along the auditory processing pathway in the formation of abstract sound representations with respect to hierarchical as well as dynamic and distributed processing models. Whereas most fMRI studies on categorical sound processing employed speech sounds, the emphasis of the current review lies on the contribution of empirical studies using natural or artificial sounds that enable separating acoustic and perceptual processing levels and avoid interference with existing category representations. Finally, we discuss the opportunities of modern analyses techniques such as multivariate pattern analysis (MVPA) in studying categorical sound representations. With their increased sensitivity to distributed activation changes—even in absence of changes in overall signal level—these analyses techniques provide a promising tool to reveal the neural underpinnings of perceptually invariant sound representations.

## Sound perception—more than time-frequency analysis

Despite major advances in the past years to unravel the functional organization principles of the auditory system, the neural processes underlying sound perception are still far from being understood. Complementary research in animals and humans has revealed the properties of responses of neurons and neuronal populations along the auditory pathway from the cochlear nucleus to the cortex. Current knowledge on the neural representation of the spectrotemporal features of the incoming sound is such that the sound spectrogram can be accurately reconstructed from neuronal population responses (Pasley et al., 2012). Yet, the precise neural representation of the acoustic sound features alone cannot explain sound perception fully. In fact, how a sound is perceived may be invariant to changes of its acoustic properties. Unless the context in which a sound is repeated is absolutely identical to the first encounter—which is rather unlikely under natural circumstances—recognizing a sound is not trivial, given that the acoustic properties of the two repetitions may not entirely match. Obviously, this poses an extreme challenge to the auditory system. To maintain processing efficiency, acoustically different sounds must be mapped onto the same perceptual representation. Thus, an essential part of sound processing is the reduction or perceptual categorization of the vast diversity of spectrotemporal events into meaningful (i.e., behaviorally relevant) units. However, despite the ease with which humans generally accomplish this task, the detection of relevant and invariant information in the complexity of the sensory input is not straightforward. This is also reflected in the performance of artificial voice and speech recognition systems for human-computer interaction, that is far below that of humans, which is mainly due to the difficulty of dealing with the naturally occurring variability in speech signals (Benzeguiba et al., 2007). In humans, the need for perceptual abstraction in everyday functioning manifests itself in pathological conditions such as the autism spectrum disorder (ASD). Next to their susceptibility to more general cognitive deficits in abstract reasoning and concept formation (Minshew et al., 2002), individuals with ASD tend to show enhanced processing of detailed acoustic information while processing of more complex and socially relevant sounds such as speech may be diminished (reviewed in Ouimet et al., 2012).

**Figure 1:**
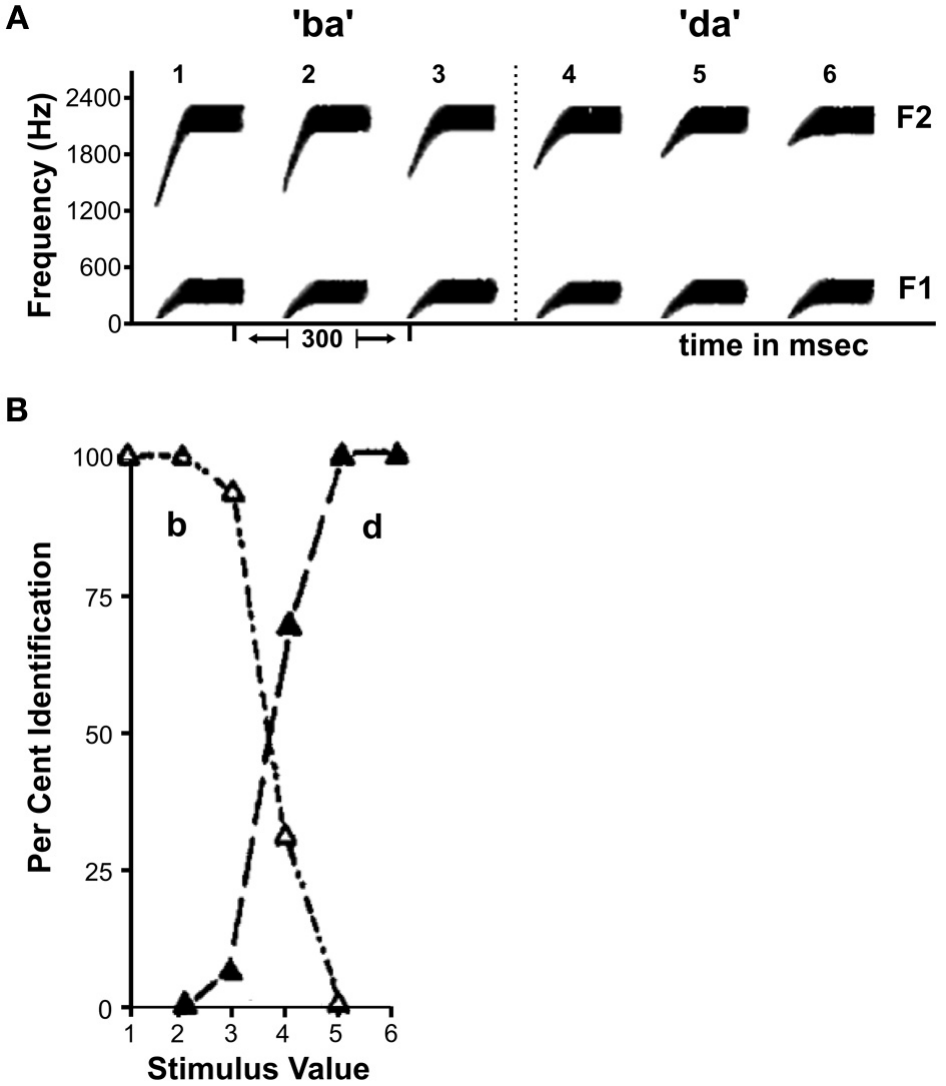
**Illustration of the sensory-perceptual transformation of speech sounds**. **(A)** Schematic representation of spectral patterns for the continuum between the phonemes /b/ and /d/. F1 and F2 reflect the first and second formant (i.e., amplitude peaks in the frequency spectrum). **(B)** Phoneme identification curves corresponding to the continuum in A. Curves are characterized by relatively stable percepts within a phoneme category and sharp transitions in between. Figure adapted from Liberman et al. (1957).

Speech sounds have been widely investigated in the context of sensory-perceptual transformation as they represent a prominent example of perceptual sound categories that comprise a large number of acoustically different sounds. Interestingly, there is not a clear boundary between two phoneme categories such as /b/ and /d/: the underlying acoustic features vary smoothly from one category to the next (Figure 1A). Remarkably though, if people are asked to identify individual sounds randomly taken from this spectrotemporal continuum as either /b/ or /d/ their percept does not vary gradually as suggested by the sensory input. Instead, the sounds from the first portion of the continuum are robustly identified as /b/, while the sounds from the second part are perceived as /d/ with an abrupt perceptual switch in between (Figure 1B). Performance on discrimination tests further suggests that people are fairly insensitive to the underlying variation of the stimuli within one phoneme category, mapping various physically different stimuli onto the same perceptual object (Liberman et al., 1957). At the category boundary, however, the same extent of physical difference is perceived as a change in stimulus identity. This difference in perceptual discrimination also affects speech production, which strongly relies on online monitoring of auditory feedback. Typically, a self-produced error in the articulation of a speech sound is instantaneously corrected for if, e.g., the output vowel differs from the intended vowel category. An acoustic deviation of the same magnitude and direction may however be tolerated if the produced sound and the intended sound fall within the same perceptual category (Niziolek and Guenther, 2013). This suggests that the within-category differences in the physical domain are perceptually compressed to create a robust representation of the phoneme category while between-category differences are perceptually enhanced to rapidly detect the relevant change of phoneme identity. This phenomenon is termed “Categorical Perception” (CP, Harnad, 1987) and has been demonstrated for stimuli from various natural domains apart from speech, such as music (Burns and Ward, 1978), color (Bornstein et al., 1976; Franklin and Davies, 2004) and facial expressions of emotion (Etcoff and Magee, 1992), not only for humans but also for monkeys (Freedman et al., 2001, 2003), chinchillas (Kuhl and Miller, 1975), songbirds (Prather et al., 2009), and even crickets (Wyttenbach et al., 1996). Thus, the formation of discrete perceptual categories from a continuous physical signal seems to be a universal reduction mechanism to deal with the complexity of natural environments.

Several recent reviews have discussed the neural representation of sound categories in auditory cortex (AC) and the role of learning-induced plasticity (e.g., Nourski and Brugge, 2011; Spierer et al., 2011). The emphasis of the current review lies on recent empirical studies using natural or artificial sounds and experimental paradigms that enable separating acoustic and perceptual processing levels and avoid interference with existing category representations (such as for speech). Additionally, we discuss the opportunities of modern analyses techniques such as multivariate pattern analysis (MVPA) in studying categorical sound representations.

## The role of experience in the formation of perceptual categories

While CP has been demonstrated many times for a large variety of stimuli, the mechanisms underlying this phenomenon remain debated. Even for speech, which has most widely been investigated, the relative contribution of innate processes and learning in the formation of phoneme categories is not completely resolved. Despite the striking consistency of perceptual phoneme boundaries across different listeners, behavioral evidence suggests that those boundaries are malleable depending on the context in which the sounds are perceived (Benders et al., 2010). Additionally, cross-cultural studies have shown that language learning influences the discriminability of speech sounds, such that phonemes in one particular language are only perceived categorically by speakers of that language and continuously otherwise (Kuhl et al., 1992). Similarly, lifelong (e.g., musical training) as well as short-term experience both affect behavioral processing—and neural encoding (see below)—of relevant speech cues, such as pitch, timber and timing (Kraus et al., 2009). In support of the claim that speech CP can be acquired through training stand experimental learning studies that successfully induced discontinuous perception of a non-native phoneme continuum through elaborate category training (Myers and Swan, 2012). Nevertheless, even after extensive training, non-native phoneme contrasts tend to remain less robust than speech categories in the native language. Apart from the age of acquisition, the complexity of the learning environment and in particular the offered stimulus variability during category learning seems to affect the ability to discriminate novel phonetic contrasts (Logan et al., 1991). A prevalent theory for the formation of speech categories in particular is the motor theory of speech perception (Liberman and Mattingly, 1985). This theory claims that speech sounds are categorized based on the distinct motor commands for the vocal tract used for pronunciation. Further fueled by the discovery of mirror neurons, the theory still has its proponents (for review see Galantucci et al., 2006), however, today, it is disputed in its strict form in which speech processing is considered special, as the recruitment of the motor system for sound identification has been demonstrated for various forms of non-speech action-related sounds (Kohler et al., 2002). Furthermore, accumulating evidence indicates that CP can be induced by learning for a variety of non-speech stimulus material (e.g., simple noise sounds, Guenther et al., 1999 and inharmonic tone complexes, Goudbeek et al., 2009). The use of artificially constructed categories for studying CP has the advantage that the physical distance between neighboring stimuli can be controlled such that the similarity ratings of within- or between-category stimuli can be attributed to true perceptual effects, rather than the metrics of the stimulus dimensions. Nevertheless, one should bear in mind that the long-term exposure to statistical regularities of the acoustics of natural sounds might exert a lasting influence on the formation of new sound categories. In support of this claim, Scharinger et al. (2013b) revealed a strong preference for negatively correlated spectral dimensions typical for speech and other natural categories when participants learned to categorize novel auditory stimuli. In line with this behavioral documentation in humans, a recent study in rodent pups demonstrated the proneness of auditory receptive fields to the systematics of the acoustic environment shaping the tuning curves of cortical neurons. Most importantly, these neuronal changes were shown to parallel an increase in perceptual discrimination of the employed sounds, which points to a link between (early) neuronal plasticity and perceptual discrimination ability (Köver et al., 2013). In sum, these experiments demonstrated that the perceptual abilities could be modified by learning and experience, while the role of pre-existing (i.e., innate) neural structures and their early adaptation in critical phases of maturation might play a vital role.

## Neural representations of perceptual sound categories

Behavioral studies have been complemented with research on the neural implementation of perceptual sound categories. Forming new sound categories or assigning a new stimulus to an existing category requires the integration of bottom-up stimulus driven information with knowledge from prior experience and memory as well as linking this information to the appropriate response in case of an active categorization task. Different research lines have highlighted the contribution of neural structures along the auditory pathway and in the cortex to this complex and dynamic process.

Functional neuroimaging studies employing natural sound categories such as voices, speech, and music have located object-specific processing units in higher level auditory areas in the superior temporal lobe (Belin et al., 2000; Leaver and Rauschecker, 2010). Particularly, native phoneme categories were shown to recruit the left superior temporal sulcus (STS) (Liebenthal et al., 2005) and the activation level of this region seems to correlate with the degree of categorical processing (Desai et al., 2008). While categorical processes in the STS were documented by further studies, the generalization to other sound categories beyond speech remains controversial, given that the employed stimuli were either speech sounds or artificial sounds with speech-like characteristics (Leech et al., 2009; Liebenthal et al., 2010). Even if speech sounds are natural examples of the discrepancy between sensory and perceptual space, the results derived from these studies may not generalize to other categories, as humans are processing experts for speech (similar to faces) even prior to linguistic experience (Eimas et al., 1987). In addition, regions in the temporal lobe were shown to retain the sensitivity to acoustic variability within sound categories, while highly abstract phoneme representations (i.e., invariant to changes within one phonetic category) appear to depend on decision-related processes in the frontal lobe (Myers et al., 2009). These results are highly compatible with those from cell recordings in rhesus monkey (Tsunada et al., 2011). Based on the analysis of single-cell responses to human speech categories, the authors suggest that “a hierarchical relationship exists between the superior temporal gyrus (STG) and the ventral PFC whereby STG provides the ‘sensory evidence’ to form the decision and ventral PFC activity encodes the output of the decision process.” Analog to the two-stage hierarchical processing model in the visual domain (Freedman et al., 2003; Jiang et al., 2007; Li et al., 2009), the set of findings reviewed above suggests that processing areas in the temporal lobe only constitute a preparatory stage for categorization. Specifically, the model proposes that the tuning of neuronal populations in lower-level sensory areas is sharpened according to the category-relevant stimulus features, forming a task-independent reduction of the sensory input (but see below for a different view on the role of early auditory areas). In case of an active categorization task, this information is projected to higher-order cortical areas in the frontal lobe. The predominant recruitment of the prefrontal cortex (PFC) during early phases of category learning (Little and Thulborn, 2005) and in the context of an active categorization task (Boettiger and D'Esposito, 2005; Husain et al., 2006; Li et al., 2009) support the concept that it plays a major role in rule learning and attention-related processes modulating lower-level sound processing rather than being the site of categorical sound representations *per se*.

Categorical processing does however not exclusively proceed along the auditory “what” stream. To study the neural basis of CP, Raizada and Poldrack (2007) measured fMRI while subjects listened to pairs of stimuli taken from a phonetic /ba/-/da/ continuum. Responses in the supramarginal gyrus were significantly larger for pairs that included stimuli belonging to different phonetic categories (i.e., crossing the category boundary) than for pairs with stimuli from a single category. The authors interpreted these results as evidence for “neural amplification” of relevant stimulus difference and thus for categorical processing in the supramarginal gyrus. Similar analyses showed comparatively little amplification of changes that crossed category boundaries in low-level auditory cortical areas (Raizada and Poldrack, 2007). Novel findings revived the motor theory of categorical processing: Chevillet et al. (2013) provide evidence that the role of the premotor cortex (PMC) is not limited to motor-related processes during active categorization, but that the phoneme-category tuning of premotor regions may essentially facilitate also more automatic speech processes via dorsal projections originating from pSTS. While this automatic motor route is probably limited to processing of speech and other action-related sound categories, the diversity of the categorical processing networks documented in the above cited studies demonstrates that there is not a single answer to where and how sound categories are represented. The role that early auditory cortical fields play in the perceptual abstraction from the acoustic input remains a relevant topic of current research. A recent study from Nelken's group indicated that neurons in the cat primary auditory area convey more information about abstract auditory entities than about the spectro-temporal sound structure (Chechik and Nelken, 2012). These results are in line with the proposal that neuronal populations in primary AC encode perceptual abstractions of sounds (or *auditory objects*, Griffiths and Warren, 2004) rather than their physical make up (Nelken, 2004). Furthermore, research from Scheich's group has suggested that sound representations in primary AC are largely context- and task- dependent and reflect memory-related and semantic aspects of actively listening to sounds (Scheich et al., 2007). This suggestion is also supported by the observation of semantic/categorical effects within early (~70 ms) post-stimulus time windows in human auditory evoked potentials (Murray et al., 2006).

Finding empirical evidence for abstract categorical representations in low-level auditory cortex in humans, however, remains challenging as it requires experimental paradigms and analysis methods that allow disentangling the perceptual processes from the strong dependence of these auditory neurons on the physical sound attributes. Here, carefully controlled stimulation paradigms in combination with fMRI pattern decoding (see below) could shed light on the matter. For example, Staeren et al. (2009) were able to dissociate perceptual from stimulus-driven processes by controlling the physical overlap of stimuli within and between natural sound categories. They revealed categorical sound representations in spatially distributed and even overlapping activation patterns in early areas of human AC. Similarly, studies employing fMRI-decoding to investigate the auditory cortical processing of speech/voice categories have put forward a “constructive” role of early auditory cortical networks in the formation of perceptual sound representations (Formisano et al., 2008; Kilian-Hütten et al., 2011a; Bonte et al., 2014).

Crucially, studying context-dependence and plasticity of sound representations in early auditory areas may help unraveling their nature. For example, Dehaene-Lambertz et al. (2005) demonstrated that even early low-level sound processing is susceptible to top-down directed cognitive influences. In a combination of fMRI and electrophysiological measures, they showed that identical acoustic stimuli were processed in a different fashion, depending on the “perceptual mode” (i.e., whether participants perceived the sounds as speech or artificial whistles).

This literature review illustrates that in order to understand the neural mechanisms underlying the formation of perceptual categories, it is necessary to (1) carefully separate perceptual from acoustical sound representations, (2) distinguish between lower-level perceptual representations and higher-order or feedback-guided decision- and task-related processes and also (3) avoid interference with existing processing networks for familiar and overlearned sound categories.

## Learning and plasticity

Most knowledge about categorical processing in the brain is derived from experiments employing speech or other natural (e.g., music) sound categories. While providing important insights about the neural representations of familiar sound categories, these studies lack the potential to investigate the mechanisms underlying the transformation from acoustic to more abstract perceptual representations. Sound processing must however remain highly plastic beyond sensitive periods early in ontogenesis to allow efficient processing adapted to the changing requirements of the acoustic environment.

Studying these rapid experience-related neural reorganizations requires controlled learning paradigms of new sound categories. With novel, artificial sounds, the acoustic properties can be controlled, such that physical and perceptual representations can be decoupled and interference with existing representations of familiar sound categories can be avoided (but see Scharinger et al., 2013b). A comparison of pre- and post-learning neural responses provides information about the amenability of sound representations along different levels of the auditory processing hierarchy to learning-induced plasticity. Extensive research by Fritz and colleagues has provided convincing evidence for learning-induced plasticity of cortical receptive fields. In ferrets that were trained on a target (tone) detection task, a large proportion of cells in primary AC showed significant changes in spectro-temporal receptive field (STRF) shape during the detection task, as compared with the passive pre-behavioral STRF. Relevant to the focus of this review, in two-thirds of these cells the changes persisted in the post-behavior passive state (Fritz et al., 2003, see also Shamma and Fritz, 2014). Additionally, recent results from animal models and human studies have revealed evidence for similar cellular and behavioral mechanisms for learning and memory in the auditory brainstem (e.g., Tzounopoulos and Kraus, 2009).

Learning studies further provide the opportunity to look into the interaction of lower-level sensory and higher-level association cortex during task- and decision-related processes (De Souza et al., 2013). In contrast to juvenile plasticity, which is mainly driven by bottom-up input, adult learning is supposedly largely dependent on top-down control (Kral, 2013). Thus, categorical processing after short-term plasticity induced by temporary changes of environmental demands might differ from the processes formed by early-onset and long-term adaptation to speech stimuli. Even though there is evidence that with increasing proficiency in category discrimination, neural processing of newly learned speech sounds starts to parallel that of native speech (Golestani and Zatorre, 2004), a discrepancy between ventral and dorsal processing networks for highly familiar native sound categories and non-native or artificial sound categories respectively has been suggested by recent work (Callan et al., 2004; Liebenthal et al., 2010, 2013). This difference potentially limits the generalization to native speech of findings derived from studies employing artificial sound categories.

Several studies have examined the changes in the neural sound representations underlying the perceptual transformations induced by category learning. A seminal study with gerbils demonstrated that learning to categorize artificial sounds in the form of frequency sweeps resulted in a transition from a physical (i.e., onset frequency) to a categorical (i.e., up vs. down) sound representation already in the primary AC (Ohl et al., 2001). In contrast to the traditional understanding of primary AC as a feature detector, this finding implicates that sound representations at the first cortical analysis stage are more abstract and prone to plastic reorganization imposed by changes in environmental demands. In fact, sound stimuli have passed through several levels of basic feature analyses before they ascend to the superior temporal cortex (Nelken, 2004). Thus, as discussed above, sound representations in primary AC are unlikely to be faithful copies of the physical characteristics. Even though the involvement of AC in categorization of artificial sounds has also been demonstrated in humans (Guenther et al., 2004), conventional subtraction paradigms typically employed in fMRI studies lack sufficient sensitivity to demarcate distinct categorical representations. Due to the large physical variability within categories and the similarity of sounds straddling the category boundary, between-category contrasts often do not reveal significant results (Klein and Zatorre, 2011). Furthermore, the effects of category learning on sound processing as demonstrated in animals were based on changes in the spatiotemporal activation pattern without apparent changes in response strength (Ohl et al., 2001; Engineer et al., 2014). Using *in vivo* two-photon calcium imaging in mice, Bathellier et al. (2012) have convincingly shown that categorical sound representations—which can be selected for behavioral or perceptual decisions—may emerge as a consequence of non-linear dynamics in local networks in the auditory cortex (Bathellier et al., 2012, see also Tsunada et al., 2012 and a recent review by Mizrahi et al., 2014).

In human neuroimaging, these neuronal effects that do not manifest as changes in overall response levels may remain inscrutable to univariate contrast analyses. Also, fMRI designs based on adaptation, or more generally, on measuring responses to stimulus pairs/sequences (e.g., as in Raizada and Poldrack, 2007) do not allow excluding generic effects related to the processing of sound sequences or potential hemodynamic confounds, as the reflection of neuronal adaptation/suppression effects in the fMRI signals is complex (Boynton and Finney, 2003; Verhoef et al., 2008).

**Figure 2:**
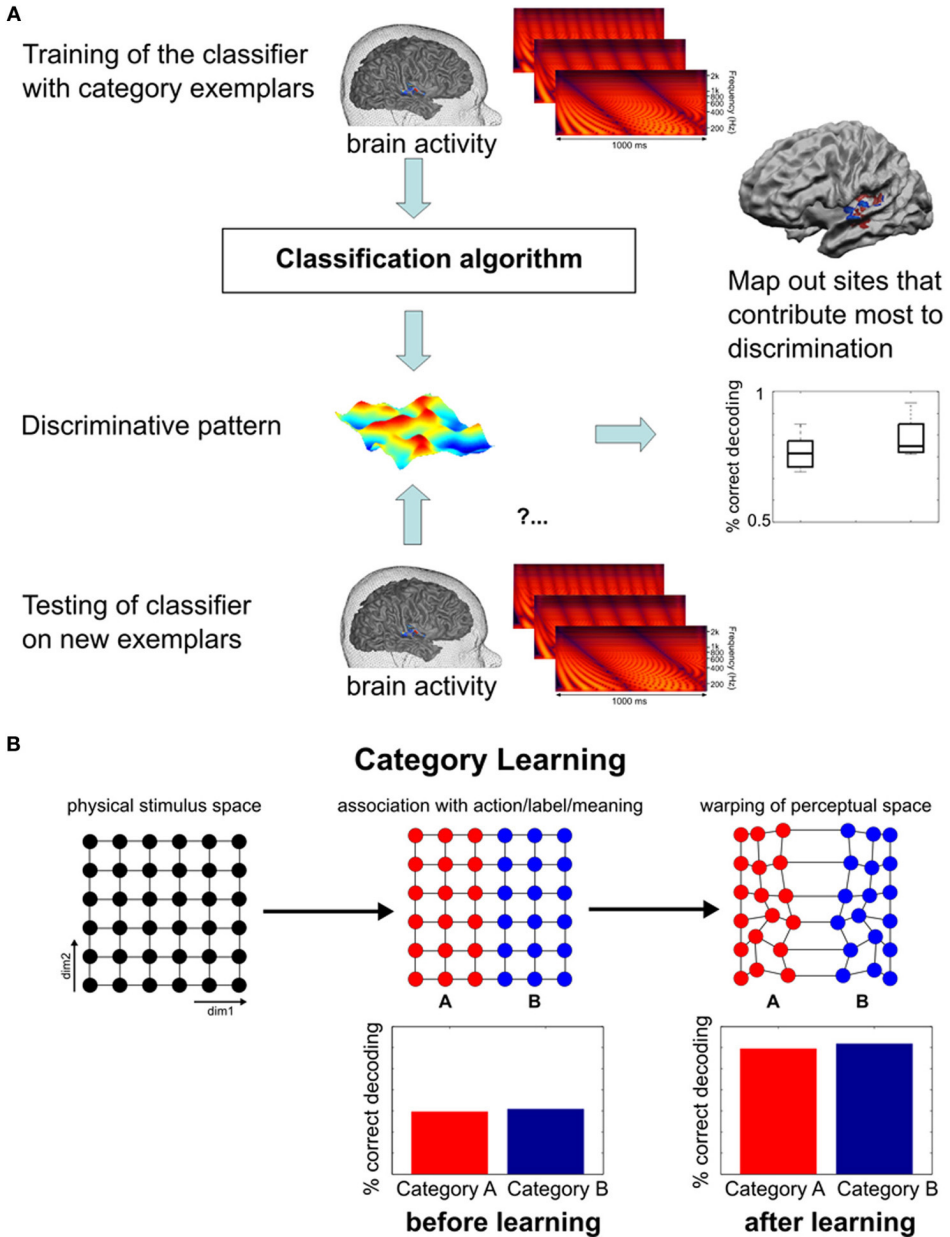
**Functional MRI pattern decoding and rationale for its application in the neuroimaging of learning**. **(A)** General logic of fMRI pattern decoding (Figure adapted from Formisano et al., 2008). Trials (and corresponding multivariate responses) are split into a training set and a testing set. On the training set of data, response patterns that maximally discriminate the stimulus categories are estimated; the testing set of data is then used to measure the correctness of discrimination of new, unlabeled trials. For statistical assessment, the same analysis is repeated for different splits of learning and test sets. **(B)** Schematic representation of the perceptual (and possibly neural) transformation from a continuum to a discrete categorical representation. The first plot depicts an artificial two-dimensional stimulus space without physical indications of a category boundary (exemplars are equally spaced along both dimensions). During learning, stimuli are separated according to the relevant dimension, irrespective of the variability in the second dimension. Lasting differential responses for the left and right half of the continuum eventually lead to a warping of the perceptual space in which within-category differences are reduced and between-category differences enlarged. Graphics inspired by Kuhl (2000). Thus, in cortical regions where (sound) categories are represented, higher fMRI-based decoding accuracy of responses to stimuli from the two categories is expected *after learning*.

**Figure 3:**
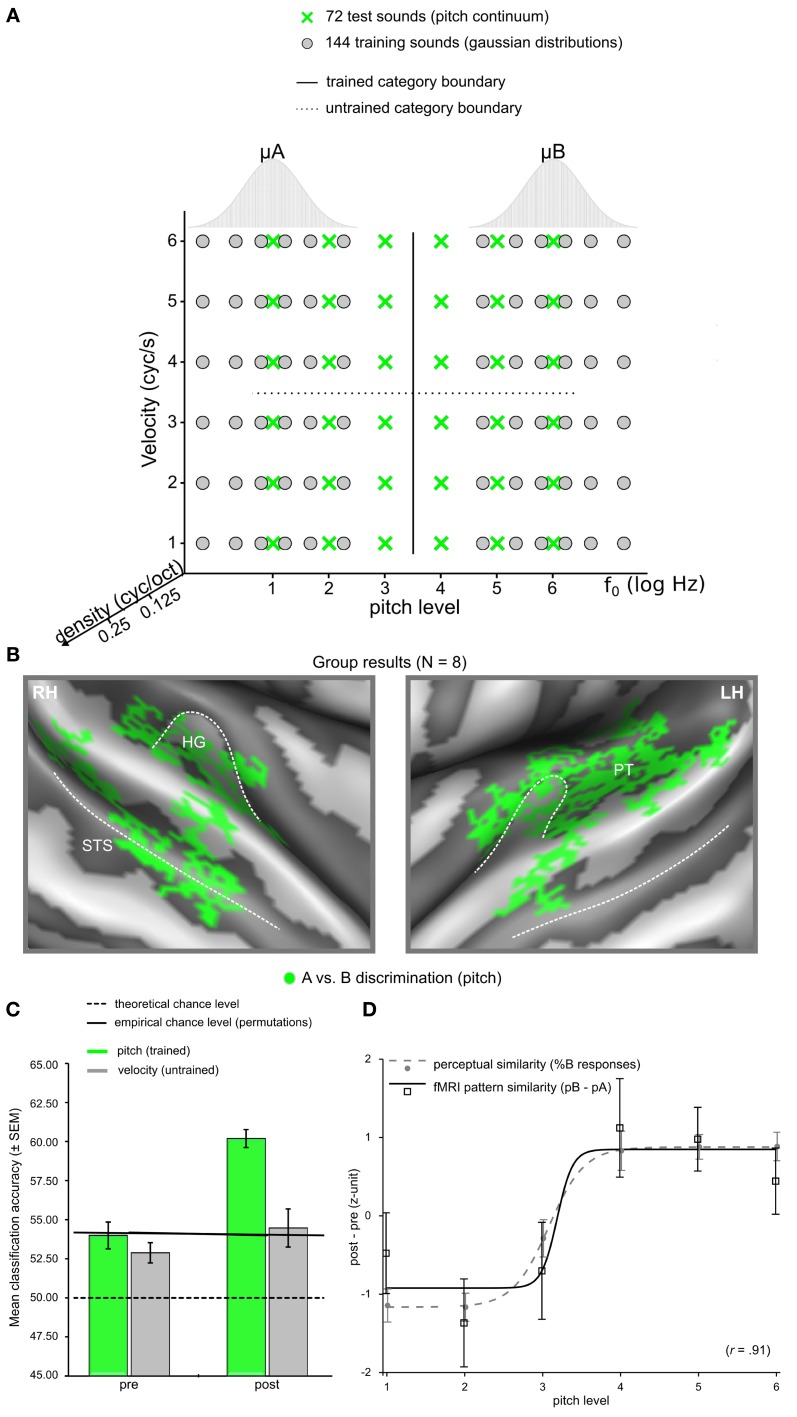
**Representation of the study by Ley et al. (2012)**. **(A)** Multidimensional stimulus space spanning the two categories A and B. **(B)** Group discrimination maps based on the post-learning fMRI data for the trained stimulus division (i.e., “low pitch” vs. “high pitch”), displayed on an average reconstructed cortical surface after cortex-based realignment. **(C)** Average classification accuracies based on fMRI data prior to category training and after successful category learning for the two types of stimulus space divisions (trained vs. untrained) and the respective trial labeling. **(D)** Changes in pattern similarity and behavioral identification curves. After category learning, neural response patterns for sounds with higher pitch (pitch levels 4, 5, 6) correlated with the prototypical response pattern for class B more strongly than class A, independent of other acoustic features. The profile of these correlations on the pitch continuum closely reflected the sigmoid shape of the behavioral category identification function.

Modern analyses techniques with increased sensitivity to spatially distributed activation changes in absence of changes in overall signal level provide a promising tool to decode perceptually invariant sound representations in humans (Formisano et al., 2008; Kilian-Hütten et al., 2011a) and detect the neural effects of learning (Figure 2). Multivariate pattern analysis (MVPA) employs established classification techniques from machine learning to discriminate between different cognitive states that are represented in the combined activity of multiple locally distributed voxels, even when their average activity does not differ between conditions (see Haynes and Rees, 2006; Norman et al., 2006; Haxby, 2012 for tutorial reviews). Recently, Ley et al. (2012) demonstrated the potential of this method to trace rapid transformations of neural sound representations, which are entirely based on changes in the way the sounds are perceived induced by a few days of category learning (Figure 3). In their study, participants were trained to categorize complex artificial ripple sounds, differing along several acoustic dimensions into two distinct groups. BOLD activity was measured before and after training during passive exposure to an acoustic continuum spanned between the trained categories. This design ensured that the acoustic stimulus dimensions were uninformative of the trained sound categorization such that any change in the activation pattern could be attributed to a warping of the perceptual space rather than physical distance. After successful learning, locally distributed response patterns in Heschl's gyrus (HG) and its adjacency became selective for the trained category discrimination (pitch) while the same sounds elicited indistinguishable responses before. In line with recent findings in rat primary AC (Engineer et al., 2013), the similarity of the cortical activation patterns reflected the sigmoid categorical structure and correlated with perceptual rather than physical sound similarity. Thus, complementary research in animals and humans indicate that perceptual sound categories are represented in the activation patterns of distributed neuronal populations in early auditory regions, further supporting the role of the early AC in abstract and experience-driven sound processing rather than acoustic feature mapping (Nelken, 2004). It is noteworthy that these abstract categorical representations were detectable despite passive listening conditions. This is an important detail, as it demonstrates that categorical representations are (at least partially) independent of higher-order decision or motor-related processes. Furthermore, it suggests that some preparatory (i.e., multipurpose) abstraction of the physical input happens at the level of the early auditory cortex.

The mechanisms of neuroplasticity underlying category learning and the origin of the categorical organization of sound representations in the auditory cortex are still quite poorly understood and deserve further investigation. Hypotheses are primarily derived from perceptual learning studies in animals. These studies show that extensive discrimination training may elicit reorganization of the auditory cortical maps, selectively increasing the representation of the behaviorally relevant sound features (Recanzone et al., 1993; Polley et al., 2006). This suggests that environmental and behavioral demands lead to changes of the auditory tuning properties of neurons such that more neurons are tuned to the relevant features to achieve higher sensitivity in the relevant dimension. This reorganization is mediated by synaptic plasticity, i.e., the strengthening of neuronal connections following rules of Hebbian learning (Hebb, 1949; for recent review, see Caporale and Dan, 2008). Passive learning studies suggest that attention is not necessary for sensory plasticity to occur (Watanabe et al., 2001; Seitz and Watanabe, 2003). However, in contrast to the mostly unequivocal sound structure used for perceptual learning experiments, learning to categorize a large number of sounds differing along multiple dimensions requires either sound distributions indicative of the category structure (Goudbeek et al., 2009) or a task including response feedback in order to extract the relevant and category discriminative sound feature. This selective enhancement of features requires some top-down gating mechanism. Attention can act as such a filter, increasing feature saliency (Lakatos et al., 2013) by selectively modulating the tuning properties of neurons in the auditory cortex, eventually leading to a competitive advantage of behaviorally relevant information (Bonte et al., 2009, 2014; Ahveninen et al., 2011). As a consequence, more neural resources would be allocated to the behaviorally relevant information at the expense of information that is irrelevant for the decision. The adaptive allocation of neural resources to diagnostic information after category learning is supported by evidence from monkey electrophysiology (Sigala and Logothetis, 2002; De Baene et al., 2008) and human imaging, showing decreased activation for prototypical exemplars of a category relative to exemplars near the category boundary (Guenther et al., 2004). This idea of categorical sound representations being sparse or parsimonious is also compatible with fMRI observations by Brechmann and Scheich (2005), showing an inverse correlation of auditory cortex activation and performance in an auditory categorization task. The recent discovery of a positive correlation between gray matter probability in parietal cortex and the optimal utilization of acoustic features in a categorization task (Scharinger et al., 2013a) provides further evidence for the crucial role of attentional processes in feature selection necessary for category learning. Reducing the representation of a large number of sounds too few relevant features presents an enormous processing advantage. It facilitates the read-out of the categorical pattern due to the pruned data structure and limits the neural resources by avoiding redundancies in the representation according to the concept of sparse coding (Olshausen and Field, 2004).

To date, there are several models for describing the neural circuitry between sensory and higher-order attentional processes mediating learning-induced plasticity. Predictive coding models propose that the dynamic interaction between bottom-up sensory information and top-down modulation by prior experience shapes the perceptual sound representation (Friston, 2005). This implies that categorical perception would arise from the continuous updating of the internal representation during learning to incorporate all variability present within a category, with the objective of reducing the prediction error (i.e., the difference between sensory input and internal representation). Consequently, lasting interaction between forward driven processing and backward modulation could induce synaptic plasticity and result in an internal representation that correctly matches the categorical structure and therefore optimally guides correct behavior also beyond the scope of the training period. The implementation of these Bayesian processing models rests on fairly hierarchical structures consisting of forward, backward and lateral connections entering different cortical layers (Felleman and Van Essen, 1991; Hackett, 2011). According to the Reverse Hierarchy Theory (Ahissar and Hochstein, 2004), category learning would be initiated by high-level processes involved in rule-learning, controlling via top-down modulation selective plasticity at lower-level sensory areas sharpening the responses according to the learning rule (Sussman et al., 2002; Myers and Swan, 2012). In accordance with this view, attentional modulation involving a fronto-parietal network of brain areas appears most prominent during early phases of learning, progressively decreasing with expertise (Little and Thulborn, 2005; De Souza et al., 2013). Despite recent evidence for early sensory-perceptual abstraction mechanisms in human auditory cortex (Murray et al., 2006; Bidelman et al., 2013), it is crucial to note that the reciprocal information exchange between higher-level and lower-level cortical fields happens very fast (Kral, 2013) and even within the auditory cortex, processing is characterized by complex forward, lateral and backward microcircuits (Atencio and Schreiner, 2010; Schreiner and Polley, 2014). Therefore, the origin of the categorical responses in AC is difficult to determine unless the response latencies and laminar structure are carefully investigated.

## Crossmodal plasticity—considerations for future studies

Considering that sound perception strongly relies on the integration of information represented across multiple cortical areas, simultaneous input from the other sensory modalities presents itself as a major source of influence on learning-induced plasticity of sound representations. In fact, there is compelling behavioral evidence that the human perceptual system integrates specific, event-relevant information across auditory and visual (McGurk and MacDonald, 1976) or auditory and tactile (Gick and Derrick, 2009) modalities and that mechanisms of multisensory integration can be shaped through experience (Wallace and Stein, 2007). Together, these two facts predict that visual or tactile contexts during learning have a major impact on perceptual reorganization of sound representations.

Promising insights are provided by behavioral studies showing that multimodal training designs are generally superior to unimodal training designs (Shams and Seitz, 2008). The beneficial effect of multisensory exposure during training may last beyond the training period itself reflected in increased performance after removal of the stimulus from one modality (for review, see Shams et al., 2011). This effect has been demonstrated even for brief training periods and arbitrary stimulus pairs (Ernst, 2007), promoting the view that short-term multisensory learning can lead to lasting reorganization of the processing networks (Kilian-Hütten et al., 2011a,b). Given the considerable evidence for response modulation of auditory neurons by simultaneous non-acoustic events and even crossmodal activation of the auditory cortex in absence of sound stimuli (Calvert et al., 1997; Foxe et al., 2002; Fu et al., 2003; Brosch et al., 2005; Kayser et al., 2005; Pekkola et al., 2005; Schürmann et al., 2006; Nordmark et al., 2012), it is likely that sound representations at the level of AC are also prone to influences from the visual or tactile modality. Animal electrophysiology has suggested different laminar profiles for tactile and visual pathways in the auditory cortex indicative for forward and backward directed input respectively (Schroeder and Foxe, 2002). Crucially, the quasi-laminar resolution achievable with state-of-art ultra-high field fMRI (Polimeni et al., 2010) provides new possibility to systematically investigate—in humans—the detailed neurophysiological basis underlying the influence of non-auditory input on sound perception and on learning induced plasticity in sound representations in the auditory cortex.

## Conclusion

In recent years, the phenomenon of perceptual categorization has stimulated a tremendous amount of research on the neural representation of perceptual sound categories in animals and humans. Despite this large data pool, no clear answer could yet be found on where abstract sound categories are represented in the brain. Whereas animal research provides increasing evidence for complex processing abilities of early auditory areas, results from human studies tend to promote more hierarchical processing models in which categorical perception relies on higher order temporal and frontal regions. In this review, we discussed this apparent discrepancy and illustrated the potential pitfalls attached to research on categorical sound processing. Separating perceptual and acoustical processes possibly represents the biggest challenge. In this respect, it is crucial to note that many “perceptual” effects, demonstrated in animal studies, did not manifest as changes in overall signal level. Recent research has shown that while these effects may remain inscrutable to univariate contrast analyses typically employed in human neuroimaging, modern analysis techniques—such as fMRI-decoding—is capable of unraveling perceptual processes in locally distributed activation patterns. It is also becoming increasingly evident that in order to grasp the full capacity of auditory processing in low-level auditory areas, it is necessary to consider its susceptibility to context and task, flexibly adapting its processing resources according to the environmental demands. In order to bring the advances from animal and human research closer together, future approaches on categorical sound representations in humans are likely to require an integrative combination of controlled stimulation designs, sensitive measurement techniques (e.g., high field fMRI) and advanced analysis techniques.

### Conflict of interest statement

The authors declare that the research was conducted in the absence of any commercial or financial relationships that could be construed as a potential conflict of interest.
